# Phylogenetic analysis of human rhinoviruses collected over four successive years in Sydney, Australia

**DOI:** 10.1111/irv.12404

**Published:** 2016-08-09

**Authors:** Vigneswary M. Ratnamohan, Frank Zeng, Linda Donovan, Chandini R. MacIntyre, Jen Kok, Dominic E. Dwyer

**Affiliations:** ^1^ Centre for Infectious Diseases and Microbiology Laboratory ServicesInstitute of Clinical Pathology and Medical ResearchPathology WestWestmead HospitalWestmeadNSWAustralia; ^2^ School of Public Health and Community MedicineUniversity of New South WalesKensingtonNSWAustralia; ^3^ Marie Bashir Institute for Infectious Diseases and BiosecurityWestmead HospitalUniversity of SydneyWestmeadNSWAustralia; ^4^ Centre for Research Excellence in Critical InfectionsWestmead HospitalUniversity of SydneyWestmeadNSWAustralia

**Keywords:** human rhinovirus, influenza‐like illness, molecular typing, phylogeny

## Abstract

**Background:**

Human rhinoviruses (HRV) cause a wide spectrum of disease, ranging from a mild influenza‐like illness (ILI) to severe respiratory infection. Molecular epidemiological data are limited for HRV circulating in the Southern Hemisphere.

**Objectives:**

To identify the species and genotypes of HRV from clinical samples collected in Sydney, Australia, from 2006 to 2009.

**Methods:**

Combined nose and throat swabs or nasopharyngeal aspirates collected from individuals with ILI were tested for HRV using real‐time reverse‐transcriptase polymerase chain reaction (RT‐PCR). Sequencing data of 5′UTR and VP4/VP2 coding regions on RT‐PCR‐positive specimens were analysed.

**Results:**

Human rhinoviruses were detected by real‐time PCR in 20.9% (116/555) of samples tested. Phylogenetic analysis of 5′UTR and VP4/VP2 on HRV‐positive samples was concordant in the grouping of HRV A and B species but not HRV C species. Eighty per cent (16/20) of sequences that grouped as HRV C in the VP4/VP2 tree clustered as HRV A, alongside some previously described C strains as subspecies C/A. Discordant branching was seen within HRV A group: two sequences clustering as A in the VP4/VP2 tree branched within the C/A subspecies in the 5′UTR tree, and one sequence showed identity to different HRV A strains in the two genes. The prevalence of HRV C and C/A species was greater in paediatric compared to adult patients (47.9% vs 25.5%, *P *= .032).

**Conclusion:**

Human rhinoviruses are a common cause of respiratory infections, and HRV C is present in the Southern Hemisphere. Sequencing of multiple HRV regions may be necessary to determine exact phylogenetic relationships.

## 1 Introduction

Human rhinoviruses (HRV) are a diverse virus group, currently known to contain 167 serotypes.^1^ Along with human enteroviruses (HEV), HRV belong to the *Picornaviridae* family,^2^ although they are phylogenetically unrelated to HEV despite similarities in genome organization and structure. Human rhinoviruses are generally associated with the common cold and mild upper respiratory infections,^3^ but can also cause severe respiratory infections in immunocompromised hosts (including lung and hematopoietic stem cell transplant recipients) or patients with chronic pulmonary diseases.^4^, ^5^, ^6^, ^7^ Unlike respiratory syncytial virus (RSV) and influenza viruses, HRV can cause respiratory illness throughout the year, but peak incidence occurs in early autumn and spring in temperate climates.^6^

Early molecular analyses of the HRV capsid protein coding regions clustered different serotypes into two distinct species, HRV A and HRV B.^8^ In 2007, a new HRV genetic variant (subsequently designated HRV C) was identified in patients with severe pneumonia from the United States of America (USA), Germany, Hong Kong, Australia and China.^9^, ^10^, ^11^, ^12^, ^13^ Severe respiratory disease and asthma exacerbations in children were observed.^11^, ^14^ HRV C has been further divided into two subspecies, HRV Cc and HRV Ca.^15^ HRV C strains are difficult to grow in cell lines known to support the growth of other rhinoviruses,^11^ although two HRV C isolates have been propagated in nasal epithelial cell cultures.^16^

There are limited molecular epidemiological data on HRV circulating in the Southern Hemisphere, including Australia. This study aimed to identify the species and genotypes of HRV from clinical samples collected in Sydney, Australia, over four consecutive years by analysing the nucleotide homology in the 5′UTR, VP4 and part of the VP2 capsid protein coding regions.

## 2 Materials and Methods

### 2.1 Clinical samples

Combined nose and throat swabs (NTS) or nasopharyngeal aspirates (NPA) collected from individuals with an influenza‐like illness (ILI) were tested for HRV as described below. The specimens were from three separate sources over four consecutive years: (i) as part of a study assessing the efficacy of face masks in reducing household transmission of respiratory viruses from children to their parents from 2006 to 2008 (n = 268);^17^ (ii) emergency department (ED) or hospitalized patients who were negative for RSV, influenza A and B viruses, parainfluenza viruses 1–3, adenoviruses and human metapneumovirus using indirect immunofluorescence antigen testing in 2007 (n = 132); and (iii) ED patients or outpatients who were tested by respiratory virus real‐time reverse‐transcriptase polymerase chain reaction (RT‐PCR) in 2009 (n = 155).

### 2.2 Primers and probes

Primers used to amplify all picornaviruses,^18^ the HRV‐specific probes 5′UTR and VP4‐VP2 gene sequencing primers are listed in Table 1. The RT‐PCR probe sequences were designed to include most HRV sequences available in GenBank^®^ in 2007, and sequences generated from the present study.

**Table 1:** HRV‐specific primers and probes used. The probe is based on the reverse strand, and the position number is based on HRV 14 (GenBank^®^ accession number X01087)

PCR	Sequence—name	Position	Reference
Real‐time PCR	For 5′ gcccctgaatgyggctaa—Rhi3A	441	37
	Rev 5′ gaaacacggacacccaaagta—Rhi4B	553	37
	Probe 5′FAMtggtcccrtcccgcamttgc—BHQ‐Rhi1		In‐house
	Probe 5′FAMccrtcccrsaattgctcrttacgac—BHQ‐Rhi2	516	In‐house
Sequencing 5′UTR	For 5′ garcaagyactyctgtywccccgg—EV140	176	33
	Rev 5′ acacggacacccaaagtagtcggttcc—EV170	565	33
Sequencing VP4‐VP2	For 5′ gcccctgaatgyggctaa—Rhi3A	441	37
	Rev ggtaayttccaccaccancc—EVVP4	1079	38

### 2.3 RNA extraction

RNA was prepared directly from combined NTS or NPA using Roche High Pure RNA kits (Roche, Mannheim, Germany).

### 2.4 Real‐time reverse‐transcriptase PCR (RT‐PCR)

The cDNA was reverse‐transcribed from 10 μL of specimen RNA using 100 units of SuperScript III reverse transcriptase (Invitrogen, Carlsbad, CA, USA) and 4 μL used for real‐time PCR as described previously.^19^ Amplification was performed in glass capillaries on the LightCycler^®^ 2.0 System (Roche Diagnostics GmbH, Mannheim, Germany) in a final volume of 20 μL with 0.5 μmol L^−1^ of each primer, 0.2 μmol L^−1^ of each probe and 4 μL of Roche FastStart hybridization Master Plus probes (Roche Diagnostics GmbH, Mannheim, Germany). The TaqMan real‐time PCR assay conditions consisted of denaturation (95°C for 10 minutes) and amplification (40 cycles of 95°C for 10 seconds, 58°C for 15 seconds, 72°C for 12 seconds).

### 2.5 Sequencing PCR

Human rhinoviruses RT‐PCR‐positive samples were then tested using UTR primers EV140 and EV170 to amplify a 395‐bp product.^20^ Where RNA was available, samples were amplified with primers RHI3A and EVP4R, generating a 638‐bp fragment that included part of 5′UTR, all of VP4 and part of VP2 regions.^18^, ^21^ Both PCRs used 1 unit of AmpliTaq Gold^®^ DNA Polymerase (Applied Biosystems^®^, Life Technologies, New Jersey, USA) and its supplied buffer with 4 mmol L^−1^ of MgCl_2_, 0.2 mmol L^−1^ dNTPS, 0.2 μmol L^−1^ of the forward and reverse primers and 4 μL of cDNA in a volume of 50 μL. PCR cycling conditions with primers EV140 and EV170 had an initial 10‐minute denaturation at 95°C, 40 amplification cycles (94°C for 1 minute, 57°C for 40 seconds, 72°C for 1.40 minutes) and 5‐minute final extension at 72°C. The PCR conditions for primers RHI3A and EVP4R were similar, except that annealing was at 61°C and the cycle number was 50.

### 2.6 Sequencing reaction and analysis

PCR amplicons were purified using the QIA PCR purification kit (Qiagen GmbH, Hilden, Germany) and sequenced with the respective primer in both directions in an ABI‐3100 Prism Genetic Analyzer using the BigDye Terminator version 3.1 sequencing kit (Applied Biosystems^®^, New Jersey, USA). >A homology search was carried out for the 93 sequences generated with NCBI BLAST^®^. Sequences of non‐ATCC prototype strains and the nearest fully characterized HRV types, but not partial coding sequences (cds), in GenBank^®^ were included in the construction of the phylogenetic tree. Phylogenetic analysis was performed on the 5′UTR and VP4/VP2 regions using the Geneious 6.1.8 software (Biomatters Limited, Auckland, New Zealand). Maximum‐likelihood (ML) trees with 1000 bootstraps were constructed,^22^ using the HKY85 model and selecting Coxsackie A11 as the outgroup sequence.

### 2.7 Submission to GenBank^®^

Ninety‐three sequences were submitted to GenBank^®^, 28 with 5′UTR alone and 65 with both 5′UTR and VP4/VP2. Samples and GenBank^®^ accession numbers are shown in Tables 2 and 3. Clinical samples and study samples are denoted as CS‐year and MS‐year, respectively.

**Table 2:** Species of HRV‐positive samples as determined by the three ML trees Samples that showed different branching are indicated. Strains not shown in the figures are A67‐FJ445149, A61‐FJ445144, A31‐FJ445126, A47‐JN837692, A98‐FJ445173, A28‐JN798580, A65‐JF781504, C45‐JN837686, C‐JF317015, C26‐JX193796, C‐JF317015 and C17‐JN815244

Sample ID with nearest identity to HRV strain	HRV species in 5′UTR analysis	HRV species in VP4/VP2 analysis	HRV species in 5′UTR/VP4/VP2 analysis
CS07‐7—A24	A	A	A
CS09‐5—A24	A	A	A
CS09‐22—A24	A	A	A
CS09‐3—A33	A	A	A
CS09‐12—A33	A	A	A
●CS09‐2—A67	A branches with HRV89	A‐	A
MS07‐20—A57	A	A	A
MS07‐21—A57	A	A	A
CS09‐6—A21	A	A	A
CS09‐17—A21	A	A	A
CS09‐21—A21	A	A	A
MS06‐4—A61	A	A	A
MS06‐6—A61	A	A	A
CS09‐15—A41	A	A	A
MS08‐8—A55	A	A	A
CS09‐11—A58	A	A	A
CS09‐26—A58	A	A	A
MS06‐2—A89	A	A	A
CS09‐28—A8	A	A	A
CS07‐9—1B	A	A	A
CS07‐12—1B	A	A	A
CS07‐13—1B	A	A	A
CS07‐14—1B	A	A	A
CS07‐18—1B	A	A	A
MS08‐6—A43	A	A	A
CS09‐4—A43	A	A	A
MS07‐16—A31	A	A	A
CS09‐13—A31	A	A	A
CS09‐20—A47	A	A	A
CS07‐24—A56	A	A	A
CS09‐23—A98	A	A	A
MS08‐2—A15	A	A	A
MS08‐3—A15	A	A	A
CS07‐6—A15	A	A	A
CS09‐27—A15	A	A	A
CS07‐4—A22	A	A	A
CS09‐1—A23	A	A	A
CS09‐8—A23	A	A	A
MS07‐1—KF543880	A	A	A
●MS06‐3—A12	C/A	A	A
CS07‐2—A20	A	A	A
MS07‐23—A20	A	A	A
MS07‐2—A28	A	A	A
●MS07‐18—A65	C/A	A	A
CS09‐14—B48	B	B	B
MS07‐3—C‐EU840952	C/A	C	C/A
MS07‐4—C‐EU840952	C/A	C	C/A
MS07‐5—C‐EU840952	C/A	C	C/A
MS07‐13—C‐EU840952	C/A	C	C/A
MS07‐19—C‐EU840952	C/A	C	C/A
MS06‐7—C	C	C	C
MS08‐5—C	C	C	C
MS07‐14—C	C	C	C
MS07‐9—C	C	C	C
MS06‐8—C‐EF077280	C/A	C	C/A
CS07‐1—C‐EF077280	C/A	C	C/A
MS08‐1—C‐EF077280	C/A	C	C/A
MS07‐6—C‐EF077280	C/A	C	C/A
MS08‐4	C/A	C	C/A
CS09‐25	C/A	C	C/A
CS07‐8	C/A	C	C/A
MS07‐15—C‐DQ875932	C/A	C	C/A
CS09‐24—C‐DQ875932	C/A	C	C/A
MS07‐7—C‐JQ994498	C/A	C	C/A
MS07‐17—C‐JQ994498	C/A	C	C/A

**Table 3:** Prevalence of HRV subtypes according to age groups

Samples	HRV‐positive in age group (below 10 y)	HRV‐positive in age group (10 y or above)
Mask study (MS06, MS07, MS08) (age unknown for two samples)	27 samples10 A, 9 C/A, 7 C and 1 B	11 samples7 A, 4 C/A, 0 C and 0 B
Clinical samples (CS07)	16 samples8 A, 7 C/A, 0 C and 1 B	9 samples3 A, 4 C/A, 0 C and 2 B
Clinical samples (CS09)	6 samples4 A, 0 C/A, 0 C and 2 B	22 samples16 A, 3 C/A, 0 C and 3 B

## 3 Results

### 3.1 rt‐pcr

Rhinoviruses were detected by RT‐PCR (with Ct between 19 and 35) in 116/555 (20.9%) samples (43/268 [16.0%] from 2006 to 2008, 34/132 [25.8%] in 2007 and 39/155 [25.2%] in 2009). Age distribution and gender were not recorded in all the subjects that provided specimens. There were 141 children and 125 adults (2 not known) in the study from 2006 to 2008.^17^ Of 43 subjects with confirmed HRV, 29 were children (mean age 25 months, median age 12 months), 12 were adults (mean age 33 years, median age 32 years) and two were of unknown age. Of 34 subjects with HRV in 2007, 22 were children (mean age 10.9 months, median age 6.5 months) and 12 were adults (mean age 59.6 years, median age 54 years). Of 39/155 subjects with HRV in 2009, 9/22 tested were children (mean age 40 months, median age 36 months) and 30/133 tested were adults (mean 39 years, median 42 years).

### 3.2 Sequencing PCR

One hundred and two samples had RNA available for sequencing; 94 samples were amplified using primers EV140 and EV170, and sequencing reactions were successful in 93. Sequencing PCR with primers RHI3A and EVP4R (for part of the 5′UTR, entire VP4 and part of VP2 regions) was successful in 65/93 samples; 19 were negative and nine were insufficient for this PCR. Sixty‐five specimens had sequences of approximately 900 bp, covering part of the 5′UTR, entire VP4 and approximately 232 bp of the VP2 region.

### 3.3 Phylogenetic analysis

Using BLAST^®^, nearly all the sequences showed >98% similarity to one or more HRV sequences with partial cds available in GenBank^®^. These were approximately 390–420 bp and did not cover the full sequence from 5′UTR to VP2 of our study samples, and were not included in the construction of the ML tree.

**Figure 1:**
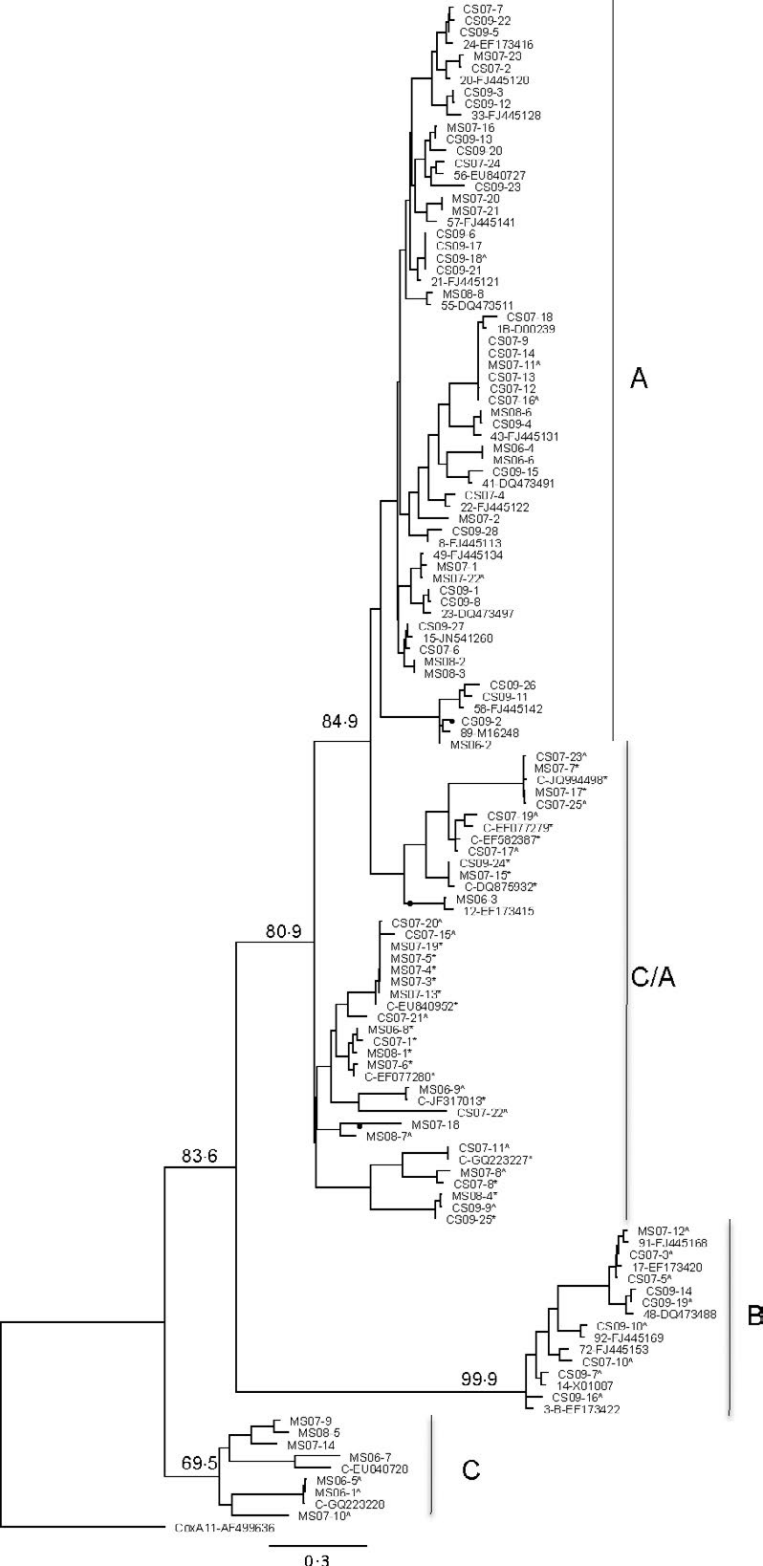
Maximum‐likelihood tree of the 5′UTR showing relationships of clinical strains, newly described strains and prototype strains of HRV. The ML tree was constructed using PHYML within Geneious Pro 6.1.8 (HKY model and 1000 bootstraps), with Coxsackie virus A11 (GenBank accession number AF499636) as the outgroup. Bootstrap values are shown for the major branches. Samples with asterisk (*) denote strains that grouped as species C in analysis of the VP4/VP2 protein coding regions, but as C/A in the 5′UTR region. Discordant branches in the VP4/VP2 and 5′UTR sequences are indicated by ●

**Figure 2:**
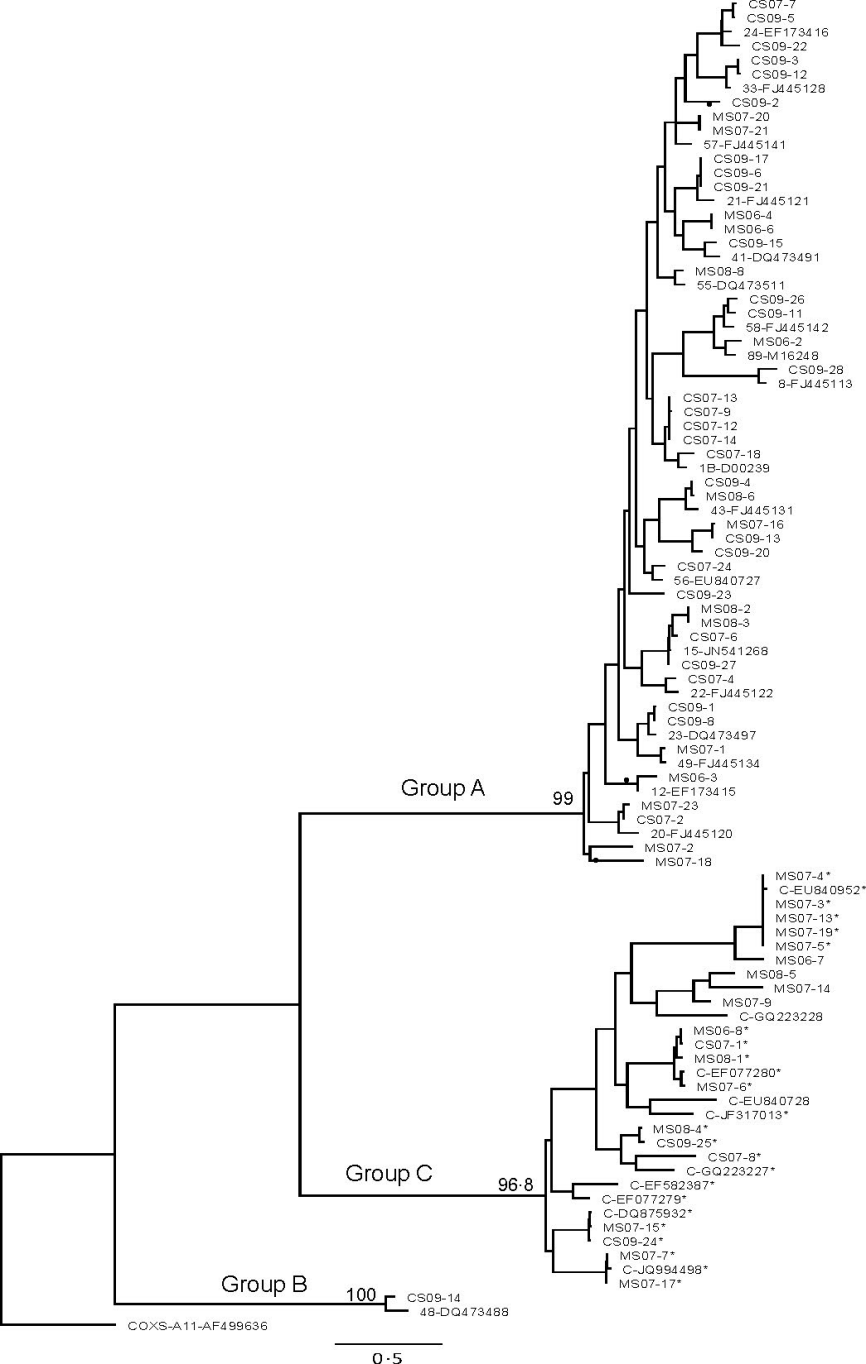
Maximum‐likelihood tree of the VP4/VP2 coding region, showing relationships of clinical strains, newly described strains and prototype strains of HRV (constructed as described for Figure 1). Samples with asterisk (*) denote strains that grouped as C in the VP4/VP2 region, but grouped with HRV A species. Samples with only 5′UTR sequences are denoted by ^. Discordant branches in the VP4/VP2 and 5′UTR sequences are indicated by ●

**Figure 3:**
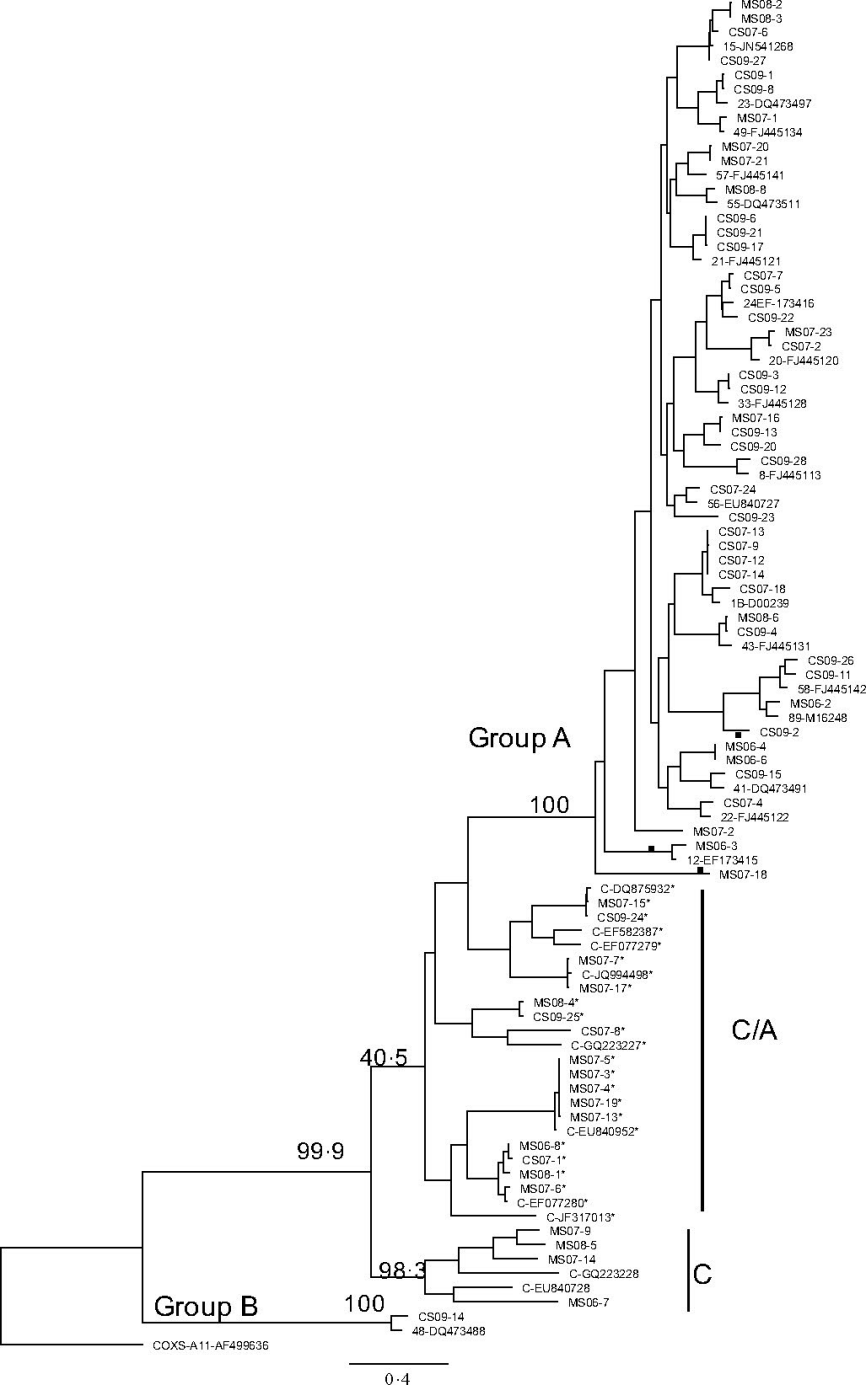
Maximum‐likelihood tree of the 5′UTR/VP4/VP2 region, showing relationships of clinical strains, newly described strains and prototype strains of HRV (constructed as described for Fig. 1). Samples with asterisk (*) denote strains that grouped as C in the VP4/VP2 region, but grouped with HRV A species in 5′UTR analysis. Discordant branches in the VP4/VP2 and 5′UTR sequences are indicated by ●

As the data size was large, it was not possible to include each one of the HRV prototype sequences in the tree construction. Figure 1 shows the ML tree for the 5′UTR for all 93 sequences, and Figs 2 and 3 show the ML trees, respectively, of VP4/VP2 and 5′UTR/VP4/VP2 regions of the 65 samples.

Table 2 shows the HRV species as determined by the ML trees of 5′UTR, VP4/VP2 and 5′UTR/VP4/VP2 regions; samples that grouped as C in VP4/VP2 but as A in 5′UTR and 5′UTR/VP4/VP2 are denoted as C/A. Serotypes and their GenBank^®^ accession numbers are shown in Tables S4 and S5, respectively (supplementary section).

### 3.4 5′UTR

Figure 1 shows the ML tree constructed using 93 sequences: 65 samples with both 5′UTR and VP4/VP2s and 28 with 5′UTR only, as denoted with a caret (^). More reference strains of HRV B species were included in the 5′UTR analysis. Characterized reference strains and samples that grouped as HRV C in the VP4/VP2 regions but as HRV A on the 5′UTR ML tree are marked with an asterisk (*).

Of the 28 samples with 5′UTR alone available, 17, 8 and 3 grouped as HRV A, B and C, respectively. Of the 17 HRV A samples, 12 HRV C samples were incorrectly grouped as HRV A. The 12 samples showed identity to previously reported C strains: CS07‐20 and CS07‐15 with C‐EU840952; MS06‐9 with C‐JF317013; CS07‐23 and CS07‐25 with C‐JQ994498; CS07‐17 with C‐EF‐582387; CS07‐19 with C‐EF077279; CS07‐11 with C‐GQ223227; MS07‐8, CS07‐21 and CS07‐22, respectively, with C17‐JN815244, C‐JF781505 and C‐GU219984 (not shown in figures). Sample CS09‐9 had no close identity.

For the 65 samples with both 5′UTR and VP4/VP2 sequences, grouping was similar for most samples that segregated into HRV A and B species as seen in the VP4/VP2 gene, but this was not the case with the HRV C group. All clinical samples that clustered in group A maintained the closest identity to the same reference strains as seen in VP4/VP2, except sample CS09‐2.

In comparing the phylogeny between VP4/VP2 and 5′UTR, the grouping of HRV C samples was different. Four of 20 samples in group C with VP4/VP2 analysis also clustered in group C in the 5′UTR along with reference strain C‐EU840728; 16 of the 20 HRV C samples grouped as HRV A, along with reference strains C‐X2‐EF077280, C‐EU840952, C‐DQ875932, C‐JQ994498, C‐EF077279, C‐EF582387 and C‐GQ223227. Four clades that grouped as C species in the VP4/VP2 analysis segregated into the A genogroup in the 5′UTR analysis (Figs 1 and 2).

Discordant branching was seen in the following and indicated by a circle (●) in Figs 1, 2 and 3. Two branches that segregated as A in VP4/VP2 tree formed part of two major branches that grouped as C/A in the UTR tree. Sample MS06‐3 and HRV A12 (EF173415) clustered in a clade more related to C‐DQ875932 (subspecies Ca). The two samples showed between 80% and 82% ID to C‐DQ875932 strain and samples in that cluster and similar identity to A15 and its cluster in the UTR tree, but in the VP4/VP2 tree the two samples showed 61% identity to C‐DQ875932 and lower to other C strains and 62% and 80% identity to A 15 and 1B, respectively.

Two samples MS07‐18 (97% homology to A65‐JF781504) and MS08‐7 (closest homology to A71‐JX025555) grouped in a major branch with known strains of HRV Ca subspecies (78%–83% identity to C‐EF077280 and 74% to 1B). In VP4/VP2, the identity for MS07‐18 was 67.2% to EF077280, but maintained around 75% to HRV A viruses like 1B, 55 and 33 reference strains. A VP4/VP2 sequence was not available for MS08‐7.

Another sample (CS09‐2) that was closest to A67 (not shown) in VP4/VP2 branched separately and clustered with A89 along with sample MS06‐2. CS09‐2 showed 77.7% relatedness to A89 in the VP4/VP2 region and greater than 95% in the UTR region.

Samples with sequences that clustered with more than 99% identity (six samples with C‐EU840952, four samples with C‐JQ994498, six samples with A‐1B‐D00239, four samples with A21‐FJ445121; Fig. 2) were all collected from the same year.

In the two families with presumed household transmission of HRV, samples MS06‐4 and MS06‐6 collected from mother and child shared the same HRV A strain. Similarly, MS07‐3, MS07‐4 and MS07‐5 collected from both parents and child shared the same HRV C strain.

### 3.5 VP4/VP2 coding region

The 65 sample sequences segregated into three phylogenetically distinct species: 44 (67.7%) HRV A, 20 (30.8%) HRV C and one (1.5%) HRV B. Several clades were represented within HRV A and C (Fig. 2).

Within HRV A, identity at > 86% was seen with known HRV reference strains as shown in Table 2 and Fig. 2. GenBank^®^ accession numbers that are not shown in the figures are 28‐JN798580, 65‐JF781504, 47‐JN837692, 67‐FJ445149, 98‐FJ445173, 31‐FJ445126 and 61‐FJ445144.

Of the 20 samples that grouped as HRV C species, identity at >85% was seen with strain C‐X2‐EF077280 (four samples), C‐EU840952 (five samples), C‐DQ875932 (two samples) and C‐JQ994498 (two samples) and four samples to the following reference strains not shown in the figure: C26‐JX193796, C‐JF317015 and C‐JF317015. Three samples (CS07‐8, CS09‐25 and MS08‐4) did not show close identity to any characterized HRV C strains in GenBank^®^.

### 3.6 5′UTR/VP4/VP2 region

The grouping of samples as C and CA in the 5′UTR/VP4/VP2 analysis was very similar to that seen in the 5′UTR analysis. The discordant branching pattern seen with samples MS06‐3 and HRV A12 (EF173415), MS07‐18 and MS08‐7 and CS09‐2 (indicated with ●) in the 5′UTR ML tree was not seen; it was in agreement with that seen in the VP4/VP2 ML tree.

### 3.7 Prevalence of HRV C according to age groups

In the mask study group where samples were collected from 2006 to 2008, sequences were available for 40 samples collected from 12 adults, 26 children aged 10 years or less and two of unknown age. Sequences of clinical samples collected in 2007 (CS07) were from patients presenting to ED and/or hospitalized. Sequences from clinical samples collected in 2009 (CS09) were from individuals not presenting to hospital but in the community suffering from symptoms of ILI. The distribution of the different HRV subtypes is shown in Table 3. Both HRV A and C or C/A variant were detected in higher numbers than HRV B. Human rhinoviruses C or C/A variant was detected more in the paediatric group (23/49) than in the adult group (11/42). There were 22/49 HRV A infections in the paediatric group and 26/42 in the adult group.

## 4 Discussion

Since 2007, new global HRV variants that are not closely related to any of the existing 101 HRV serotypes have been classified into a novel lineage, HRV C.^11^, ^23^, ^24^, ^25^, ^26^ Initially, this classification was based on limited sequence data (usually about 300 bp) in the 5′UTR region, but recently more HRV C full‐genome sequences have become available in GenBank^®^. Retrospective analyses show that HRV C has been circulating for nearly 20 years and now considered a third genetically distinct HRV subtype.^27^, ^28^ Palmenberg et al.^29^ reported the first complete study of all ATCC reference strains as well as some field isolates, which included 11 HRV C viruses. Analysis of sequencing data in this study included most of the newly recognized full‐length HRV C sequences, in addition to the GenBank^®^ sequences of known HRV A and B strains (which, in most cases, were not ATCC prototype strains) to which the clinical samples showed greatest homology. The few field isolates compared were closely identified with the reference types as minor variants.

In our study, the sequences grouped into three phylogenetically distinct species: A (52.6%), C/CA (37.6%) and B (9.6%). However, there was discordance between proposed phylogeny groups when sequences from the 5′UTR and VP4/VP2 coding regions were analysed. Sixteen of the 20 samples that clustered as HRV C in the VP4/VP2 ML tree segregated as A in the 5′UTR analysis, as did some of the early, well‐characterized HRV C strains from New York (EU840952 and DQ875932),^23^, ^26^ San Francisco (EF077279 and EF077280),^25^ Hong Kong Special Administrative Region (EF582387)^11^ and China (GQ223227).^15^ Twelve of 28 sequences with only UTR sequences also segregated along with the above‐mentioned reference C strains, branching in two major subgroups within HRV A. These have been labelled in this study as C/A, clustering with C strains, but grouping as A in the 5′UTR region: these formed 30% of the clinical sequences. Huang et al.^15^ reported similar clustering of field strains in their study and designated the above C strains (EU840952, DQ875932, EF077279, EF077280, GQ223227 and EF582387) as Ca, a subspecies of HRV C that clustered differently to HRV A, HRV B and HRV C in the UTR. They further showed that HRV Ca subspecies were formed from interspecies recombination in the 5′UTR region. Similar inconsistent clustering of field strains as compared to VP4/VP2 was also reported in the 3D polymerase‐coding region as well as 5′UTR region.^30^ Three discordant branching events were seen in our analyses. MS07‐18 (identity to A65) in one branch and MS06‐3 along with HRV A12 in another branch segregated as A in the VP4/VP2 analysis, but localized within the Ca subspecies in the 5′UTR analysis. Sample CS09‐2 clustered with HRV A89 with 96% relatedness in the UTR region but differed in the VP4/VP2 region. These three samples may represent recombinants as reported by Palmenberg et al. and Kim et al.^31^, ^32^

There is no designated region within the HRV genome that is known to give consistent genotyping results for HRV strains. It is also difficult to compare the findings when different groups have used different genes. The 5′UTR, VP4/VP2 and VP1 coding regions have been previously used to characterize genotypes. Kiang et al.^14^ compared both the 5′UTR and VP4/VP2 sequences of reference strains and clinical isolates (samples that showed CPE in W1‐38 cells and/or human foetal diploid lung cells) and demonstrated that the 5′UTR was discriminatory and was able identify the isolate as a single prototype strain. VP4/VP2 and VP1 regions are more variable than the conserved 5′UTR, hence the difficulty in designing universal diagnostic primers.^33^ McIntyre et al.^34^ found that while branching orders of VP1, VP4/VP2 and 3D pol trees were identical, greater than 60% of C variants in their study showed recombination with A sequences in the 5′UTR. They were able to map two recombination hot spots in the 5′UTR region. The amplification of the VP1 coding region is rather difficult, requiring culture isolates and multiple sets of primers.^26^, ^34^ The clustering of HRV C as C and C/A when 5′UTR region was included in the analysis (Figs 1 and 3) in our study may suggest that they are true C species that showed recombination with A in the 5′UTR.

In this study, the 5′UTR primers and the primers chosen to amplify the entire VP4 and partial VP2 region produced amplicons with overlapping sequences that resulted in an approximately 900‐bp continuous sequence. We used a single set of primers for each of the two PCR assays but did not use cloning, which has been used in other studies.^24^, ^35^ Most studies using the VP1 protein coding region for genetic analysis used multiple degenerate primer pairs on clinical samples or culture isolates to achieve the sequences of interest. Using only cell culture isolates rather than clinical samples to study the variation in phylogenetic relationship among field isolates may not be entirely accurate as cell lines generally favour the selection of certain virus strains, failing to detect other fastidious strains. Sequencing PCR products directly from clinical samples is reflective of a true representation, but the sensitivity of PCR primers varies.

Real‐time diagnostic PCR primers target the 5′UTR, a region with high interstrain homology, because of the ease of using a single set of primers that detect the majority of HRV strains. However, HRV strains with low copy numbers may remain undetected. In this study, the sensitivity of the 5′UTR sequencing primers and the second set of primers for the amplification of the full length of the VP4 and part of VP2 in comparison with the real‐time assay was 92.1% and 69.56%, respectively.^20^ Unlike others investigators, we did not find co‐infections by multiple HRV strains.^12^, ^36^ Two incidences of household HRV transmission were suggested by the sequences of all samples in each household showing 99% homology.

It is unclear whether the severity of infections caused by HRV C species is greater compared to HRV A and HRV B. The mask study had 2.5 times more children than adults who were positive for HRV, but the study selected children with a febrile respiratory illness, with well adult contacts at baseline; children who had specimens collected all presented to a paediatric ED. The majority of these HRV sequences were either C or the C/A variant (16/27). CS07 samples were from patients either presenting to ED or hospitalized and had an equal proportion of HRV C or C/A strains among adults and children. CS09 samples were from adults with an ILI who did not present to hospital during the A(H1N1)pdm09 influenza pandemic; HRV C/Ca infection was seen in only 13%. Overall, there was a greater representation of HRV C/Ca species in the paediatric compared to the adult group (47.9% vs 25.6%, *P *= .032, Fisher's exact test). All clinical samples were collected from patients with respiratory illness who were sick enough to warrant testing or in many cases hospitalization, but our data are insufficient to attribute clinical severity to any of the HRV species.

It is possible that HRV C or the C/A variants may cause exacerbation of respiratory infections in infants that require presentation to ED compared to HRV A or B, and this may contribute to the higher percentage of HRV C or C/A infection in the paediatric population. One of the early reports of HRV C‐QPM variant severity was from Australian samples collected in 2003 from children with lower respiratory infection.^37^ Other reports have observed the association between HRV C variants and asthmatic wheeze and severe lower respiratory infections.^10^, ^11^, ^23^, ^27^, ^38^ Xiang et al.^35^ reported that the clinical manifestations of HRV A and C are similar, and co‐infection with RSV and HRV in infants increases the severity of infection. Both HRV A and C are more virulent than HRV B in infants and HRV virulence is greater in winter, although peak infection rates occur in spring and fall.^9^

In conclusion, the present study shows that sequencing of one region alone is insufficient for determining the lineage of the HRV variants. The presence of many diverse strains has become apparent, and it is likely that more will emerge. Genotypic assignment and identification of HRV types will facilitate monitoring of emerging novel variants, and investigations into type‐associated differences in disease epidemiology, transmission and outcomes.

## Ethics Statement

Samples used in this study were not collected for this study *per se*, but as part of a study assessing the use of face mask in controlling respiratory virus transmission in households following approval by the local institutional review board or for laboratory diagnosis of patients with an influenza‐like illness. The present study does not involve the reporting of patient data, and no patient intervention occurred with the obtained results.

## Author Contributions

VMR participated in the study design, analysed the results and drafted the manuscript. FZ and LD performed nucleic acid testing. CRM was the chief investigator of the study on the use of face masks to reduce household transmission of respiratory viruses. JK had input in the preparation and editing of the manuscript. DED participated in the design of the study and was involved in manuscript editing. All authors have read and accepted the manuscript.
