# Supplementary material for: Phylogenetic analysis of human rhinoviruses collected over four successive years in Sydney, Australia
Source: Influenza Other Respir Viruses. 2016 Aug 9;10(6):493–503. doi: 10.1111/irv.12404 (PMC5059946; doi:10.1111/irv.12404)
Supplement: Supplementary file 1 [file IRV-10-493-s001.docx]

**SUPPLEMENTARY**

**Table S1. GenBank® accession numbers for study samples with 5'UTR and VP4/VP2 gene partial cds**

Nearest related strains are as in figures 1 and 2. Identity to the nearest characterised strain is for the VP4/VP2 region. Strains not shown in figures are indicated. Samples with no nearest strain indicated show close homology to partial cds (250 to 440 bases) and thus were not considered.

| **Sample ID** | **Age of patient** | **HRV species** | **Identity–nearest strain** | **GenBank® accession number** |
| --- | --- | --- | --- | --- |
| CS07-7 | 7 yrs | A | 24-EF173416 (90.7%) | KF543902 |
| CS09-5 | 56 yrs | A | 24-EF173416 (91.4%) | KF543921 |
| CS09-22 | 21 yrs | A | 24-EF173416 (89.4%) | KF543932 |
| CS09-3 | 3 yrs | A | 33-FJ445128 (93.7%) | KF543919 |
| CS09-12 | 55 yrs | A | 33-FJ445128 (93.2%) | KF543925 |
| CS09-2 | 5 yrs | A | 67-FJ445149 (93%)-  not shown | KF543918 |
| MS07-20 | 6 yrs | A | 57—EU096044 (85.8%) | KF543895 |
| MS07-21 | adult | A | 57—EU096044 (86%) | KF543896 |
| CS09-6 | 12 yrs | A | 21-FJ445121 (91.4%) | KF543922 |
| CS09-17 | 1 yr | A | 21-FJ445121 (91.4%) | KF543929 |
| CS09-21 | 48 yrs | A | 21-FJ445121 (91.4%) | KF543931 |
| MS06-4 | 4yrs | A | 61-FJ445144 (89%)–not shown | KF543876 |
| MS06-6 | 32 yrs | A | 61-FJ445144 (89%)–not shown | KF543877 |
| CS09-15 | adult | A | 41-DQ473491 (89.8%) | KF543928 |
| MS08-8 | adult | A | 55-DQ473511 (92.8%) | KF543915 |
| CS09-11 | 48 yrs | A | 58-FJ445142 (92.1%) | KF543924 |
| CS09-26 | 50 yrs | A | 58-FJ445142 (91.9%) | KF543936 |
| MS06-2 | 3 yrs | A | 89-M16248 (90.1%) | KF543874 |
| CS09-28 | 67 yrs | A | 8-FJ445113 (90.3%) | KF543938 |
| CS07-9 | 1 yr | A | 1B-D00239 (89.6%) | KF543904 |
| CS07-12 | 84 yrs | A | 1B-D00239 (90.1%) | KF543905 |
| CS07-13 | 85 yrs | A | 1B-D00239 (90.1%) | KF543906 |
| CS07-14 | 89 yrs | A | 1B-D00239 (90.1%) | KF543907 |
| CS07-18 | 1 mth | A | 1B-D00239 (91%) | KF543908 |
| MS08-6 | 9 mths | A | 43-FJ445131 (91.4%) | KF543915 |
| CS09-4 | 40 yrs | A | 43-FJ445131 (92.1%) | KF543920 |
| MS07-16 | child | A | A 31- FJ445126 (92%)-not shown | KF543891 |
| CS09-13 | 44 yrs | A | A 31- FJ445126 (91%)-not shown | KF543926 |
| CS09-20 | 52 yrs | A | 47-JN837692 (99%) –not shown | KF543930 |
| CS07-24 | 3 wks | A | 56-EU840727 (91.4%) | KF543909 |
| CS09-23 | 31 yrs | A | 98-FJ445173 (92%)–not shown | KF543933 |
| MS08-2 | adult | A | 15-JN541268 (91.6%) | KF543911 |
| MS08-3 | adult | A | 15-JN541268 (91.9%) | KF543912 |
| CS07-6 | 2 mths | A | 15-JN541268 (96.2%) | KF543901 |
| CS09-27 | 16 yrs | A | 15-JN541268 (98.9%) | KF543937 |
| CS07-4 | 9 mths | A | 22-FJ445122 (91.4%) | KF543900 |
| CS09-1 | 46 yrs | A | 23-DQ473497 (93.9%) | KF543917 |
| CS09-8 | 21 yrs | A | 23-DQ473497 (94.1%) | KF543923 |
| MS07-1 | 13 yrs | A | 49-FJ445134 (96.2%) | KF543880 |
| MS06-3 | 4 yrs | A | 12-EF173415 (92.3%) | KF543875 |
| CS07-2 | 7 mths | A | 20-FJ445120 (90.3%) | KF543899 |
| MS07-23 | 5 mths | A | 20-FJ445120 (89.2%) | KF543897 |
| MS07-2 | 9 mths | A | 28-JN798580 (99%)–not shown | KF543881 |
| MS07-18 | Not known | A | 65-JF781504 (99%)) –not shown | KF543893 |
| CS09-14 | 19 yrs | B | 48-DQ473488 (88.5%) | KF543927 |
| MS07-3 | 26 yrs | C/A | C-EU840952 (98.2%) | KF543882 |
| MS07-4 | 24 yrs | C/A | C-EU840952 (98.2%) | KF543883 |
| MS07-5 | 5 mths | C/A | C-EU840952 (98.2%) | KF543884 |
| MS07-13 | 3 yrs | C/A | C-EU840952 (98.2%) | KF543888 |
| MS07-19 | 6 yrs | C/A | C-EU840952 (98.2%) | KF543894 |
| MS06-7 | 1 yr | C | C45-JN837686 (86%)-not shown | KF543878 |
| MS08-5 | 1 yr | C | JF317015 (85%)-not shown | KF543914 |
| MS07-14 | 15 mths | C | C26-JX193796 (96%)-not shown | KF543889 |
| MS07-9 | 6 mths | C | JF317015 (95%)-not shown | KF543887 |
| MS06-8 | 1 yr | C/A | C-EF077280 (92.7%) | KF543879 |
| CS07-1 | 7 mths | C/A | C-EF077280 (92%) | KF543898 |
| MS08-1 | 7 mths | C/A | C-EF077280 (92%) | KF543910 |
| MS07-6 | 18 mths | C/A | C-EF077280 (97.2%) | KF543885 |
| MS08-4 | adult | C/A | None? –not shown | KF543913 |
| CS09-25 | 55 yrs | C/A | None? –not shown | KF543935 |
| CS07-8 | 5 mths | C/A | C17-JN815244 (87%) -not shown | KF543903 |
| MS07-15 | Not known | C/A | C-DQ875932 (98.9%) | KF543890 |
| CS09-24 | 24 yrs | C/A | C-DQ875932 (98.6%) | KF543934 |
| MS07-7 | 8 mths | C/A | C-JQ994498 (98.2%) | KF543886 |
| MS07-17 | 3 yrs | C/A | C-JQ994498 (98.2%) | KF543892 |

**Table S2. GenBank® accession numbers for study samples with only 5'UTR sequences**

Nearest strains are as in figures 1 and 2. Identity is shown to the nearest characterized strain. Strains not shown in figures are indicated.

| **Sample ID** | **Age of patient** | **Rhinovirus subtype** | **Identity–nearest strain** | **GenBank® accession number** |
| --- | --- | --- | --- | --- |
| CS09-18 | 3 yrs | A | 21-FJ445121 (97.1%) | KF543908 |
| MS07-11 | adult | A | 1B-D00239 (97.5%) | KF555328 |
| CS07-16 | 6 mths | A | 1B-D00239 (97.5%) | KF555336 |
| MS07-22 | 4 mths | A | 49-FJ445134 (99.1%) | KF555330 |
| MS08-7 | 4 yrs | A | 71-JX025555(99%)-not shown | KF555344 |
| CS07-23 | 37 yrs | C/A | C-JQ994498 | KF555342 |
| CS07-25 | 33 yrs | C/A | C-JQ994498 | KF555343 |
| CS07-19 | 5 mths | C/A | C-EF077279 (94.6%) | KF555338 |
| CS07-17 | 9 mths | C/A | C-EF582387 (98%) | KF555337 |
| CS07-20 | 54 yrs | C/A | C-EU840952 (98.3%) | KF555339 |
| CS07-15 | 2 mths | C/A | C-EU840952 (94.4%) | KF555335 |
| CS07-21 | 2 mths | C/A | C-JF781505 (99%)-not shown | KF555340 |
| MS06-9 | 28 yrs | C/A | C-JF317013 (98.9%) | KF555325 |
| CS07-22 | 2 mths | C/A | C-GU219984 (87%)-not shown | KF555341 |
| CS07-11 | 89 yrs | C/A | C-GQ223227 (99.7%) | KF555334 |
| MS07-8 | 1 yr | C/A | C17- JN815244 (99%)-not shown | KF555326 |
| CS09-9 | 30 yrs | C/A | None? –not shown | KF555346 |
| MS07-12 | 12 yrs | B | 91-EU096077 (98%) | KF555329 |
| CS07-3 | 35 yrs | B | 17-EF173420 (98%) | KF555331 |
| CS07-5 | 5 mths | B | 17-EF173420 (98%) | KF555332 |
| CS09-19 | 6 yrs | B | 48-DQ473488 (96.9%) | KF555350 |
| CS09-10 | 44 yrs | B | 92-FJ445169 (97.4%) | KF555347 |
| CS07-10 | 31 yrs | B | 72-FJ445153 (94.1%) | KF555333 |
| CS09-7 | 19 yrs | B | 14-X01087 (96%) | KF555345 |
| CS09-16 | 4 yrs | B | 3B-EF173422 (94.3%) | KF555348 |
| MS06-1 | 2 yrs | C | GQ223228 (99.2%) | KF555323 |
| MS06-5 | 1 yr | C | GQ223228 (98.9%) | KF555324 |
| MS07-10 | 5 mths | C | JN541267(97%)-not shown | KF555327 |
